# Self-Reported Sleep Duration and Its Correlates with Sociodemographics, Health Behaviours, Poor Mental Health, and Chronic Conditions in Rural Persons 40 Years and Older in South Africa

**DOI:** 10.3390/ijerph15071357

**Published:** 2018-06-28

**Authors:** Karl Peltzer, Supa Pengpid

**Affiliations:** 1 HIV/AIDS/STIs and TB (HAST), Human Sciences Research Council, Pretoria 0001, South Africa; 2 Department of Research and Innovation, University of Limpopo, Sovenga 0727, South Africa; supaprom@yahoo.com; 3 ASEAN Institute for Health Development, Mahidol University, Salaya 73170, Thailand

**Keywords:** sleep duration, health behaviour, mental health, chronic conditions, persons 40 years and older, rural South Africa, HAALSI

## Abstract

This study aims to investigate sleep duration and its association with sociodemographic, health behaviour, mental health, and chronic disease factors among rural individuals 40 years and older in South Africa. Cross-sectional data from the “Health and Aging in Africa: A Longitudinal Study of an INDEPTH community in South Africa” (HAALSI) baseline survey were analysed. Socio-demographic, clinical, health, and sleep duration data were collected. The total sample included 4725 persons 40 years and older (mean age 61.5 years, SD = 13.0, age range of 40–111 years) in one sub-district in rural South Africa. The mean sleep duration was 8.28 ± 1.93 h. Short (<7 h) and long (≥9 h) sleepers accounted for 13.1% and 40.0% of the sample, respectively. In adjusted multinomial logistic regression, greater wealth status (*p* < 0.05), inadequate fruit and vegetable consumption (*p* < 0.001), and depressive symptoms (*p* < 0.05) were associated with a higher odds and physical inactivity (*p* < 0.05) with a lower odds of short sleep duration. Being male (*p* < 0.001) and depressive symptoms (*p* < 0.001) were associated with a higher odds and being 50 to 69 years old (*p* < 0.01), having Grade 1 to 11 education (*p* < 0.05), and greater wealth status (*p* < 0.001) were associated with a lower odds of long sleep duration. In adjusted multinomial logistic regression, compared to normal sleepers, long sleepers were more likely to have myocardial infarction (*p* < 0.05). In unadjusted analysis, compared to normal sleepers, short sleepers were more likely to have cataracts (*p* < 0.05). This study found that a significant proportion of rural dwellers 40 years and older in South Africa had a short sleep duration and a high proportion had a long sleep duration. Some associations, such as depression and myocardial infarction, with short and/or long sleep duration were confirmed, while no associations were found for many chronic conditions.

## 1. Introduction

In several studies, older age has been found to be associated with a shorter sleep duration [1,2,3]. The National Sleep Foundation recommended seven to eight hours sleep for persons 65 years and older, considering better mental and physical health outcomes [4]. Short sleep duration (≤6 h) has been linked with negative health outcomes, including cardiovascular diseases, ischemic heart disease, hypertension, diabetes mellitus, poor mental health, chronic obstructive pulmonary disease (COPD), chronic gastroenteritis/peptic ulcer, multimorbidity, and mortality [5,6,7,8,9,10]. There has also been evidence that links a long sleep duration (≥9 h over a 24 h period) in adults and older adults with morbidity (e.g., hypertension, diabetes, cardiovascular disease, cerebrovascular diseases, stroke, atrial fibrillation, poor general health, poor mental health, hyperlipidemia, urolithiasis, multi-morbidity) and mortality [4,8,9,10,11,12,13]. 

Regarding studies on sleep duration in Africa, the mean sleep duration in a national sample of individuals 50 years and older in South Africa was 8.6 h [12] and in Ghana, it was 7.7 h [14]. In another study, in a sample of rural and urban community dwelling adults in Ghana, the objectively measured total sleep time was, on average, 7.6 h, which was a little lower than the average self-reported sleep time per night (7.8 h) [15]. The prevalence of short sleep (≤6 h) in a national study on persons 50 years and older in South Africa was 11.4% and the prevalence of long sleep (≥9 h) was 43.5% [12]. In the study about community dwelling adults in Ghana, the objectively measured prevalence of short sleep (≤6 h) was 29.3% and long sleep (≥9 h) was 38.4% [15].

Sociodemographic factors (older age, female sex) and lifestyle factors (inadequate fruit intake, current tobacco use, and physical inactivity) have also been found to be associated with a short sleep duration [8,9,12], and sociodemographic factors (older age, younger age, higher education) and lifestyle factors (inadequate fruit intake, current smoking, alcohol use, physical inactivity) have been associated with long sleep [8,9,12]. Investigating the association between sleep duration and chronic conditions may contribute to the understanding of mortality that is related to unhealthy sleep duration [8].

This study aims to investigate sleep duration and its association with sociodemographic, health behaviour, mental health, and chronic disease factors among rural middle-older South Africans. It was hypothesized that sleep duration differs by different health outcomes.

## 2. Materials and Methods

### 2.1. Sample and Procedure

Baseline data were analysed from the “Health and Ageing in Africa: A Longitudinal Study of an INDEPTH Community in South Africa (HAALSI) in the INDEPTH Health and Demographic Surveillance System (HDSS) site of Agincourt” in rural South Africa [16]. From the selected 6281 men and women, 5059 completed home interviews (response rate 85.9%) [16] and 4725 completed assessments of sleep duration; those with incomplete sleep duration assessment were excluded from the analysis. Trained field workers also collected blood through finger pricks and prepared dried bloodspots (DBS) from each participant who consented to blood collection. The study was conducted in 2015. Signed informed consent was obtained from all respondents prior to assessments. The study received ethical approvals from the University of the Witwatersrand Human Research Ethics Committee (ref. M141159), the Harvard T.H. Chan School of Public Health, Office of Human Research Administration (ref. C13-1608-02), and the Mpumalanga Provincial Research and Ethics Committee. [16].

### 2.2. Measures

Sleep duration was assessed with the question, “Over the past four weeks, how many hours do you think you actually slept each day?” [17] We followed a similar categorization used by previous studies and classified sleep hours into three categories: short sleep (<7 h per day), normal sleep (7–8 h), and long sleep (≥9 h per day) [18].

Sociodemographic variables included age, sex, formal education, and a wealth index in quintiles created from household characteristics and ownership of household items, livestock, and vehicles [16].

#### 2.2.1. Health Behaviour and Mental Health Variables

Current tobacco use was assessed with two questions on current smoking and current smokeless tobacco use, such as snuff, chewing tobacco, snus, and betel with tobacco [16].

Alcohol dependence was assessed with the four-item “Cut down-Annoyed-Guilty-Eye opener” (CAGE) questionnaire [19]. For example, “Have you ever felt you should cut down on your drinking?” Item responses to the CAGE are scored 0 or 1, with scores of 2 or more indicating alcohol dependence [19] (Cronbach’s alpha 0.82).

Fruit and vegetable consumption were assessed with two questions, “How many servings of fruit/vegetables do you eat on a typical day? (on any one day)” (with the help of show-cards) [16]. Inadequate fruit and vegetable consumption was classified as having less than five servings a day.

*Physical activity* was measured using the validated General Physical Activity Questionnaire (GPAQ) [20,21]. It assessed days and duration of physical activity at work, for transport, and during leisure time in a usual week. Results were classified into low, moderate, and high physical activity according to GPAQ guidelines [21]. 

Body Mass Index (BMI) was calculated from standardized body height and weight measures, and classified into underweight (<18.5 kg/m^2^), normal weight (18.5–24.9 kg/m^2^), overweight (25–29.9 kg/m^2^), and obesity (30+ kg/m^2^), using World Health Organization (WHO) criteria [22].

Depression symptoms were screened using the Center for Epidemiological Studies—Depression Scale (CES-D) eight-item questionnaire, with a cut-off of three or more symptoms signifying depressive symptoms [23] (Cronbach’s alpha 0.66).

Posttraumatic Stress Disorder (*PTSD*) was screened using a seven-item symptom scale developed by Breslau et al. [24], with scores of four or more indication caseness for PTSD symptoms (Cronbach’s alpha 0.83).

#### 2.2.2. Chronic Conditions

Hypertension was measured with three blood pressure readings (averaging the last two measurements) and classified if “systolic blood pressure was greater than or equal to 140 mmHg or diastolic blood pressure was 90 mmHg or higher, or if use of anti-hypertensive medication was reported at the time of interview” [16].

Dyslipidaemia was classified as “those who meet at least one of the following criteria: total cholesterol > 6.21 mmol/L, HDL-C < 1.19 mmol/L, LDL-C > 4.1 mmol/L, triglycerides > 2.25 mmol/L, reported ever diagnosed with high cholesterol, or if use of medication is reported at the time of interview” [16].

Diabetes was classified with “fasting glucose (defined as >8 h) >7 mmol/L (126 mg/dL) or non-fasting glucose level >11.0 mmol/L (200 mg/dL), reported ever being diagnosed with diabetes, or if use of medication is reported at the time of interview” [16]. 

Anaemia was classified as “a blood haemoglobin concentration of <13 g/dL for men or <12 g/dL for women” [25].

Stroke, angina, cataracts, heart failure, kidney disease, myocardial infarction, HIV, and tuberculosis were assessed by self-reported diagnosis [16].

Chronic bronchitis was assessed based on symptoms reported [26].

Multi-morbidity was defined as having two to 13 of the above 13 chronic conditions.

### 2.3. Data Analysis

Descriptive statistics were used to describe the sample and sleep duration levels. Associations between sociodemographic, health behaviour, and mental health variables and short and long sleep duration were calculated using unconditional multinomial logistic regression. The independent association of 13 chronic conditions with short and long sleep duration was assessed with multinomial logistic regression analysis, after adjusting for age, sex, education, wealth status, tobacco use, alcohol dependence, physical inactivity, inadequate fruit and vegetable consumption, BMI body weight, depression, and PTSD symptoms. No collinearity was detected and missing values were excluded from the analysis. *p* < 0.05 was considered significant. All statistical procedures were done with STATA software version 13.0 (Stata Corporation, College Station, TX, USA).

## 3. Results

### 3.1. Sample Characteristics 

The total sample included 4725 adults 40 years and older (mean age 61.5 years, SD = 13.0, age range of 40–111 years) in rural South Africa. Sample characteristics are described in Table 1. The mean sleep duration was 8.28 ± 1.93 h. Short and long sleepers accounted for 13.1% and 40.0% of the sample, respectively (see Table 1).

### 3.2. Chronic Conditions and Multi-Morbidity by Sleep Duration Categories

Table 2 describes the prevalence of 13 chronic conditions and multi-morbidity by sleep duration categories. The prevalence of having cataracts was significantly higher in short sleepers than normal sleepers and myocardial infarction was significantly higher in long sleepers than normal sleepers (see Table 2).

### 3.3. Factors Associated with Short and Long Sleep Duration

In adjusted multinomial logistic regression, greater wealth status, inadequate fruit and vegetable consumption, and depressive symptoms were positively, and physical inactivity negatively, associated with short sleep duration. Being male and having depressive symptoms increased the odds, and being 50 to 69 years old, having Grade 1 to 11 education, and greater wealth status decreased the odds, of long sleep duration (see Table 3). 

### 3.4. Associations between Chronic Conditions and Sleep Duration

In adjusted multinomial logistic regression, compared to normal sleepers, long sleepers were more likely to have myocardial infarction. In unadjusted analysis, compared to normal sleepers, short sleepers were more likely to have cataracts, but the association with cataracts did not persist after adjustment (see Table 4).

## 4. Discussion

The study focused on investigating the prevalence and correlates of short and long sleep duration in middle- and older-aged individuals in rural South Africa. The mean sleep duration was 8.3 h, which is similar to a previous national study among individuals 50 years and older in South Africa (8.6 h) [12], but lower than in a national study on persons 50 years and older in Ghana (7.7 h), China (7.6 h), and India (7.1 h) [25]. The prevalence of short sleep (13.1%) was similar to the national study on persons 50 years and older in South Africa (11.4%), [12] but was lower than in individuals 50 years and older in China (20.3%) and India (29.3%) [27], and the prevalence of long sleep (40.0%) was similar to the national study on persons 50 years and older in South Africa (43.5%) [12], but was higher than in individuals 50 years and older in China (25.9%) and India (14.9%) [27].

In unadjusted analysis, the prevalence of long sleep increased with age, but this was no longer the case in the adjusted analysis. There were no age differences in short sleep. Similar results were found in the national study persons 50 years and older in South Africa [12]. There were also no gender differences in short sleep, but men had a higher prevalence of long sleep than women did. Such differences were not found in the national study of persons 50 years and older South Africa [12]. In persons 50 years and older in six countries, women reported a longer mean sleep duration than men [14]. This sample consisted of rural dwellers in South Africa and it is possible that traditional gender roles lead to lower demands on men and an increase in sleeping duration. This study found that greater wealth status was associated with a higher odds of short sleep and lower odds of long sleep. It is possible that those with a better economic status work longer hours, have greater job responsibility, greater perceived stress, and more depressive symptoms, which may affect the sleep duration negatively [28,29].

Consistent with a previous systematic review [10], having depressive symptoms was associated with both short and long sleep. Since this was a cross-sectional study, we could not determine the direction of the association. Zhai et al. [10] found in their review of prospective studies that both short and long sleep are a risk for the development of depression. Contrary to expectation [8], this study found a weak negative association between physical inactivity and short sleep. This finding calls for further investigations. Some studies [8,9,12] found that various health risk behaviours were associated with short and/or long sleep, while this study only found a positive association between inadequate fruit and vegetable consumption and short sleep duration. Mechanisms that may mediate associations between short sleep and inadequate fruit and vegetable consumption may be multi-factorial [8,9]. It is possible that an inadequate diet increases the risk for chronic diseases, which in turn can affect sleep duration [30]. Unlike some previous studies [8,9], this study did not find an association between multi-morbidity and short and/or long sleep.

Regarding specific chronic conditions, this study only found an association between myocardial infarction and long sleep and in an unadjusted analysis, cataracts and short sleep, while there no associations for anaemia, angina, chronic bronchitis, diabetes, dyslipidaemia, heart failure, hypertension, kidney disease, stroke, HIV, and TB. Various studies have confirmed the relationship between cardiovascular conditions such as myocardial infarction with long sleep [4,8,9,10,11,12,13]. In a study in Ghana, sleep duration was also not associated with the prevalence of cardiovascular disorders [15]. Wang et al. [8] also did not find an association between anaemia, diabetes, and short and/or long sleep. This study did not confirm findings from previous studies on the association between hypertension, diabetes mellitus, ischemic heart disease, COPD, multimorbidity, and short sleep [5,6,7,8,9,10], and the association between hypertension, diabetes, cardiovascular disease, hyperlipidaemia, cerebrovascular diseases, multi-morbidity, and long sleep [4,8,9,11,12,13]. The lack of associations found in this study may be attributed to the low prevalence of short sleep and very high prevalence of long sleep. It could also be that rural individuals 40 years and older in this sample in South Africa use adaptive coping strategies to deal with their stress and thus, better their health and reduce the risk for cardio-metabolic disorders [6].

### Limitations of the Study

The self-reported assessment of sleep duration has its limitations. Self-reported sleep duration may be an overestimation of the actual measured sleep duration [15,31]. This may contribute in explaining the large proportion of long sleepers. Several other relevant sleep-related indicators, such as the use of hypnotic medications, shift work, and environmental and cultural factors, were not assessed in this study, which could have increased the understanding of the relationship chronic conditions and sleep duration, and should be assessed in future studies. Furthermore, this study was based on cross-sectional data in one sub-district in rural South Africa, hindering causative conclusions and generalizations beyond the study district. 

## 5. Conclusions

This study found a significant proportion with short sleep and a high proportion with long sleep among 40 years and older rural dwellers in South Africa. Potential factors associated with the risk for short and long sleep duration are related to sociodemographic and lifestyle factors. Some associations, such as depression and myocardial infarction, with short and/or long sleep duration were confirmed, while no associations were found with many chronic conditions. Further, (longitudinal) studies are need to gain a better understanding of possible associations.

## Figures and Tables

**Table 1:** Sample characteristics.

Variable	Sample	Short Sleepers	Normal Sleepers	Long Sleepers	Statistics
	*N* (%)	%	%	%	%
Sociodemographics					
All	4725	13.1	46.9	40	
Age (years)					
40–49	867 (18.3)	12.5	47.9	39.7	<0.001
50–59	1338 (28.3)	14.6	59	35.4	
60–69	1217 (25.8)	13.3	48.8	37.9	
70–79	814 (17.2)	13.4	42.3	44.3	
80 or more	489 (10.3)	8.6	39.7	51.7	
Sex					
Female	2513 (53.2)	13.3	48.5	38.2	0.024
Male	2212 (46.8)	12.8	45.1	42.1	
Formal education					
None	2124 (45.1)	11.5	43.9	44.6	<0.001
Grade 1–7	1603 (34.0)	14.8	47.8	37.4	
Grade 8–11	549 (11.6)	12	51.7	36.2	
Grade 12 or more	438 (9.3)	15.3	52.1	32.6	
Wealth index					
Poor	956 (20.2)	11.1	42.1	46.9	<0.001
Second poorest	928 (19.6)	10.9	44.4	44.7	
Medium	932 (19.7)	13	48.6	38.4	
Second richest	938 (19.9)	15.5	49	35.5	
Rich	971 (20.6)	14.8	50.4	34.8	
Health variables					
Body Mass Index (BMI) (kg/m^2^)					
<18.5	241 (5.5)	11.6	44.8	43.6	0.457
18.5 to <25	1604 (36.6)	12.2	47.3	40.5	
25 to <30	1230 (28.1)	13.9	46.6	39.5	
≥30	1310 (29.9)	13.2	49.1	37.7	
Current tobacco use	731 (15.5)	14.8	45.8	39.4	0.32
Alcohol dependence	65 (1.4)	20	38.5	41.5	0.175
Fruit and vegetable consumption (<5 servings)	4127 (87.8)	14	48.7	37.3	<0.001
Physical activity (low)	4697 (42.9)	11.2	45.3	43.5	<0.001
Depressive symptoms	771 (16.7)	13.9	41.9	44.2	0.004
PTSD symptoms	223 (4.8)	13.9	47.1	39	0.954

**Table 2:** Chronic diseases by sleep duration groups.

Variable	Total	Short Sleepers	Normal Sleepers	Long Sleepers	Statistics
	*N* (%)	%	%	%	
Anaemia	727 (17.4)	88 (12.1)	334 (45.9)	305 (42.0)	0.229
Angina	109 (2.3)	9 (8.3)	45 (41.3)	55 (50.5)	0.057
Cataract	610 (12.9)	99 (16.2)	286 (46.9)	225 (36.9)	0.028
Chronic bronchitis	70 (1.5)	6 (8.6)	30 (42.9)	34 (48.6)	0.268
Diabetes	528 (12.2)	66 (12.5)	254 (48.1)	208 (39.4)	0.907
Dyslipidaemia	1728 (43.6)	218 (12.6)	839 (48.6)	671 (38.8)	0.291
Heart failure	33 (0.7)	5 (15.2)	12 (36.4)	16 (48.5)	0.475
Hypertension	2697 (58.6)	354 (13.1)	1293 (47.9)	1050 (38.9)	0.215
Kidney disease	193 (4.1)	19 (9.8)	92 (47.7)	82 (42.5)	0.387
Myocardial infarction	20 (0.4)	2 (10.0)	4 (20.0)	14 (70.0)	0.021
Stroke	135 (2.9)	16 (11.9)	67 (49.6)	52 (38.5)	0.795
HIV	586 (12.5)	88 (15.0)	270 (46.1)	228 (38.9)	0.320
Tuberculosis	418 (8.9)	62 (14.8)	205 (49.0)	151 (36.1)	0.191
Number of chronic conditions					
0	455 (12.1)	64 (14.1)	215 (47.3)	176 (38.7)	0.511
1	1198 (31.8)	166 (13.9)	544 (45.4)	488 (40.7)	
≥2	2116 (56.1)	284 (13.4)	1028 (48.6)	804 (38.0)	

**Table 3:** Associations between sociodemographic and health factors in relation to sleep duration.

Variable	Short (<7 h) vs. Normal (7–8 h) Sleepers		Long (≥9 h) vs. Normal (7–8 h) Sleepers	
	COR	AOR	COR	AOR
Sociodemographics				
Age (years)				
40–49	1 (Reference)	1 (Reference)	1 (Reference)	1 (Reference)
50–59	1.13 (0.86, 1.47)	1.08 (0.80, 1.49)	0.85 (0.71, 1.03)	0.70 (0.56, 0.88) **
60–69	1.05 (0.80, 1.38)	1.07 (0.75, 1.51)	0.94 (0.78, 1.13)	0.70 (0.55, 0.89) **
70–79	1.22 (0.90, 1.65)	1.35 (0.91, 1.99)	1.27 (1.03, 1.56) *	0.90 (0.69, 1.18)
80 or more	0.83 (0.56, 1.24)	1.01 (0.60, 1.70)	1.57 (1.24, 1.99) ***	1.12 (0.80, 1.55)
Sex				
Female	1 (Reference)	1 (Reference)	1 (Reference)	1 (Reference)
Male	1.04 (0.87, 1.25)	1.01 (0.81, 1.27)	1.18 (1.05, 1.34) **	1.32 (1.13, 1.55) ***
Formal education				
None	1 (Reference)	1 (Reference)	1 (Reference)	1 (Reference)
Grade 1–7	1.18 (0.97, 1.45)	1.16 (0.91, 1.49)	0.77 (0.67, 0.89) ***	0.84 (0.70, 0.99) *
Grade 8–11	0.89 (0.66, 1.20)	0.74 (0.51, 1.09)	0.69 (0.56, 0.85) ***	0.69 (0.53, 0.89) **
Grade 12 or more	1.12 (0.83, 1.53)	1.11 (0.72, 1.72)	0.62 (0.49, 0.78) ***	0.78 (0.57, 1.08)
Wealth index				
Poor	1 (Reference)	1 (Reference)	1 (Reference)	1 (Reference)
Second poorest	0.90 (0.69, 1.26)	1.15 (0.80, 1.65)	0.90 (0.75, 1.10)	0.91 (0.72, 1.14)
Medium	0.71 (0.76, 1.36)	1.28 (0.90, 1.81)	0.71 (0.59, 0.86) ***	0.64 (0.51, 0.81) ***
Second richest	0.65 (0.90, 1.59)	1.41 (0.99, 2.00)	0.65 (0.54, 0.79) ***	0.68 (0.54, 0.87) **
Rich	0.62 (0.84, 1.48)	1.45 (1.01, 2.08) *	0.62 (0.51, 0.75) ***	0.63 (0.49, 0.80) ***
Health variables				
Body Mass Index (BMI) (kg/m^2^)				
18.5 to <25	1 (Reference)	1 (Reference)	1 (Reference)	1 (Reference)
<18.5	1.00 (0.64, 1.56)	0.97 (0.58, 1.63)	1.13 (0.85, 1.51)	0.99 (0.70, 1.41)
25 to <30	1.15 (0.92, 1.46)	1.23 (0.94, 1.60)	0.99 (0.84, 1.16)	1.09 (0.90, 1.31)
≥30	1.04 (0.83, 1.31)	1.10 (0.83, 1.45)	0.90 (0.77, 1.05)	1.07 (0.88, 1.30)
Current tobacco use	1.19 (0.94, 1.57)	1.28 (0.95, 1.72)	1.05 (0.85, 1.19)	0.80 (0.66, 1.01)
Alcohol dependence	1.89 (0.96, 3.71)	1.57 (0.72, 3.41)	1.27 (0.73, 2.19)	0.81 (0.41, 1.60)
Fruit and vegetable consumption (<5 servings)	1.82 (1.31, 2.52) ***	2.02 (1.36, 3.02) ***	0.98 (0.82, 1.18)	0.95 (0.76, 1.19)
Physical activity (low)	0.83 (0.69, 0.99) *	0.80 (0.64, 0.99) *	1.24 (1.09, 1.40) ***	1.12 (0.96, 1.31)
Depressive symptoms	1.22 (0.96, 1.55)	1.34 (1.01, 1.79) *	1.32 (1.12, 1.56) ***	1.41 (1.15, 1.74) ***
PTSD symptoms	1.96 (0.70, 1.59)	1.23 (0.76, 1.99)	0.99 (0.74, 1.32)	1.06 (0.74, 1.51)
Number of chronic conditions				
0	1 (Reference)	1 (Reference)	1 (Reference)	1 (Reference)
1	1.03 (0.74, 1.72)	0.99 (0.70, 1.39)	1.10 (0.87, 1.38)	0.99 (0.70, 1.44)
≥2	0.93 (0.68, 1.26)	0.86 (0.62, 1.19)	0.96 (0.77, 1.19)	0.94 (0.74, 1.19)

COR = Crude Odds Ratio; AOR = Adjusted Odds Ratio; *** *p* < 0.001; ** *p* < 0.01; * *p* < 0.05.

**Table 4:** Associations between chronic conditions and sleep duration.

Chronic Condition	Short (<7 h) vs. Normal (7–8 h) Sleepers		Long (≥9 h) vs. Normal (7–8 h) Sleepers	
	COR	AOR ^1^	COR	AOR ^1^
Anaemia	0.92 (0.72, 1.19)	0.89 (0.67, 1.17)	1.12 (0.95, 1.33)	1.10 (0.91, 1.32)
Angina	0.71 (0.35, 1.47)	0.78 (0.37, 1.64)	1.44 (0.97, 2.15)	1.51 (0.97, 2.35)
Cataract	1.29 (1.01, 1.65) *	1.24 (0.94, 1.63)	0.91 (0.76, 1.10)	0.81 (0.66, 1.00)
Chronic bronchitis	0.72 (0.30, 1.73)	0.68 (0.28, 1.67)	1.33 (0.81, 2.19)	1.21 (0.72, 2.02)
Diabetes	0.94 (0.71, 1.26)	0.90 (0.66, 1.23)	0.97 (0.80, 1.18)	0.95 (0.77, 1.18)
Dyslipidaemia	0.87 (0.72, 1.06)	0.83 (0.67, 1.02)	0.92 (0.81, 1.06)	0.91 (0.78, 1.05)
Heart failure	1.50 (0.53, 4.28)	1.22 (0.39, 3.89)	1.57 (0.84, 3.32)	1.45 (0.65, 3.25)
Hypertension	0.98 (0.81, 1.18)	0.93 (0.76, 1.14)	0.90 (0.79, 1.02)	0.87 (0.76, 1.01)
Kidney disease	0.73 (0.44, 1.21)	0.68 (0.40, 1.16)	1.05 (0.77, 1.42)	1.05 (0.76, 1.46)
Myocardial infarction	1.80 (0.33, 9.84)	2.13 (0.35, 12.95)	4.12 (1.36, 12.55) *	4.11 (1.13, 14.98) *
Stroke	0.85 (0.49, 1.48)	0.71 (0.37, 1.39)	0.91 (0.63, 1.31)	0.82 (0.52, 1.28)
HIV	1.20 (0.93, 1.55)	1.22 (0.92, 1.62)	0.99 (0.82, 1.19)	1.10 (0.90, 1.35)
Tuberculosis	1.10 (0.81, 1.48)	1.05 (0.76, 1.46)	0.85 (0.68, 1.06)	0.86 (0.68, 1.09)

COR = Crude Odds Ratio; AOR = Adjusted Odds Ratio; ^1^ Adjusted for age, sex, education, wealth status, tobacco use, alcohol dependence, physical inactivity, inadequate fruit and vegetable consumption, BMI body weight, depression, and PTSD symptoms; * *p* < 0.05.
